# Doctors and Physic

**Published:** 1860-08

**Authors:** 


### Doctors and Physic.

*Hear me, my friends! who this good banquet grace;*

*'Tis sweet to play the fool in time and place.*

POPE. HOMER. *Odys.*

"*A little nonsense now and then,*

*Is relished by the wisest men.*"

GERONTE—*It is impossible to reason better, Doctor. But dear sir. there is one thing which staggers me in your lucid explanation. I always thought till now, that the heart was on the left side and the liver on the right.*
MOCK DOCTOR—*Ay, sir, so they were formely; but we have changed all that. The College at present, sir, proceeds upon an entire new method.*
GEORNTE—*I ask your pardon, sir.*
MOCK DOCTOR—*Oh, sir! there is no harm—you are not obliged to know so much as we do.*
GERONTE—*Very true, Doctor, very true.*
MOLIERE.

1. PROVERBS AND SAYINGS ON DOCTORS' DOINGS.—"If the Doctor cures, the sun sees it; if he kills, the earth hides it." "The earth covers the mistakes of the physician." "Bleed him, and purge him; if he dies, bury him." "The doctor is often more to be feared than the disease." *Italian French Spanish.* Sir W. Hamilton said: "Medicine in the hands in which it is vulgarly dispensed is a curse to humanity rather than a blessing." Sir Astley Cooper avowed: "The science of medicine was founded on conjecture, and improved by murder." "The Doctor seldom takes physic," says the Italian. The German wit writes: "Physic does good always, if not to the patient, at least to the apothecary." The

Spaniard tells: "It is God that cures, and the doctor gets the money;" and "if you have a friend who is a doctor, take off your hat to him, and send him to the house of your enemy."—*Proverbs of all nations.*

2. THE PUZZLED DOCTOR—"The Seranes, Borden writes, "father and son, were physicians at the hospital at Montpelier. The son was a lively theorist, who knew by heart and was continually repeating, all the written documents about inflammation, just as children repeat in their silly way: 'The grasshopper has chirupped all the summer, etc.;' or, 'Mr. Crow sits perched on a tree, etc.' Serance, the father, was a good soul, who had studied under great masters. He had learned to treat fluxions of the chest with emetics, giving them at least every second day, with or without the addition of two ounces of manna; they were his great *chevaux de bataille.* I have seen him fire them off a thousand times, everywhere and to every one. The son resolved to convert his father to the fashion of the day; that is to say, to inspire him with a salutary dread of *phlogosis*, *erethismus*, and the rupture of the small vessels. The good father consequently fell into a most singular kind of indecisions: he knew not how to act. However, he held out firmly against bleeding; and when he came to a patient, he would murmer awhile, and then pass on without ordering anything. I have frequently known him apostrophise his son with earnestness, and to cry out, when he was desirous but yet afraid of administering an emetic: 'My son, you have ruined me (*Mon fil*, *m'abes gastat*)!' Never shall I forget this curious scene. I am greatly indebted to it, and so also were the patients of the Hospital. They got well without being bled, because old Serane did not like the bleeding, and without taking emetics, because young Serane had proved to his father that these remedies increased inflammation. The patients got well, and I learnt a profitable lesson. I concluded that the numerous bleedings with Seranes, the son, practised, when alone, were at least as useless as the repeated emetics to which Seranes, the father, was so much attached."—*Medical Times & Gazette.*

3. Dissecting a Quack.—"To puzzle a Philadelphia lawyer," is a common saying with Transatlantic brethren; but the following is related of the manner in which one of these gentlemen puzzled a quack, who was bringing a suit for medical services. *Counsel*—Did you treat the patient according to the most approved rules of surgery? *Witness*—Certainly, by all means, I did. *Counsel*—Did you decapitate him? *Witness*—Undoubtedly, I did that as a matter of course. *Counsel*—Did you perform the Cæsarean operation upon him? *Witness*—Why, of course; his condition required it; and it was attended with great success. *Counsel*—Did you, now, doctor, subject his person to autopsy? *Witness*—Certainly; that was the best remedy I adopted. *Counsel*—Well, then, doctor, as you first cut off the defendant's head, then ripped up his body, and afterwards dissected him, and he still survives it, I have no more to ask.—*Edin. Med. Jour.*

4. Pinel.—When Pinel undertook the management of Bicetre, the Revolution was at its height. The notorious Couthon presided over the dreaded *Commune* of Paris, and when Pinel came to him to obtain permission to remove the chains from the madmen, went himself the next day to the asylum, fearing lest in such an act there should be some hidden attempt against the Democratic Government. When he saw the madmen, he turned to Pinel and said: "Are you not mad yourself, to wish to deliver these *betes feroces* from their chains!" "No," answered Pinel, for I am certain that their chains make these wretched people thus violent." 'Do as you like,' said Couthon. From this time the good work commenced. The following day he removed the chains from fifty, and from thirty more a few days afterwards. The Academy of Medicine has adorned one of its rooms with a picture of this scene of humanity. Shortly after this we are told that Pinel was seized by some of the ruffians of the day, under the pretext of being an aristocrat, and hurried off "*a la lanterne;*" and that his life was saved by an old soldier, one Chevigne, whom he had delivered from his chains in the Bicetre, and who had become his servant.

Pinel states, in letters of his lately published, that he was enabled to live decently on what he obtained by the translation of English works. "At this moment," he says, "I am engaged in the translation of Cullen's 'Institutes of Medicine,' for which I receive one thousand francs. I would give up physic altogether, if it were necessary for me to be running about all day in the streets. I wish for only a very small practice—to see little and observe much."

*Lunatics in the Good Old Times.*—It is impossible for us to imagine the condition of lunatic asylums before the days of Pinal. Dungeons, wet and infected, without light and air, called *loges*, containing a wreched mattress, or some rotten straw strewed on the ground. Human beings, naked or covered with rags, almost always, furious, chained to each other, and shut up in these abodes of desolation and misery—actual tombs, out of which they came to be carried to their last dwelling-place. Their brutal keepers, selected from those who had been condemned to punishment, treated them like brutes, and used them most barbarously, abusing and ridiculing them, striking them cruelly, and continually fighting with them, throwing them their deficient and coarse food, keeping their water from them when thirsty, and their clothes when cold; exposing them to the jokes of visitors.—Such were the inhabitants of Bicetre at that time—wretched beings thought incurable, abandoned by their freinds, deprived of all medical treatment, pale, wasted, wallowing in their own filth, groaning under the weight of the chains which tore their wasted limbs, raving under the horrible sufferings to which they were subjected by their inhuman keepers.—*Gaz. Hebd. Med. Times & Gazette.*

5. "They manage things better in France."—The dryness and want of animation which usually illustrate debates in our own English Medical Societies, have been often cast in their teeth; it is well, therefore, to hear what a French critic thinks of some of those brilliant out bursts of eloquence which we are often inclined to envey in listening to the prosaic humdrum way of doing business which John

Bull usually indulges. "They manage these things better in France!" Let us see. Here are the general remarks, made *a propos* of the particular occasion on which M. Trousseau indulged the French Academy with an hour's burst of eloquence:

"And this leads us to a remark of more general application. Oratorical struggles in an Academy of Medicine have rather a beautiful than a good effect. They give animation, piquancy and popularity to the meeting. But people come to them to hear an orator, not for instruction. Those long brilliant discourses, which refer more *ad hominem* than *ad rem*, or which have no direct reference either to the matter or the person, are like so many beautiful solos executed on different airs. They frighten away from the discussion many a modest but stedfast man, who would speak his mind freely in private society or in his seat, but who has not the courage to mount up to a tribune still vibrating with the echoes of an eloquent peroration. We merely signalize the fact without deducing any consequence from it. There is no other way of curing the evil than by limiting the length of the discourses, but whenever this has been tried it has been given up. Generally speaking, the orators whose voices the Society thus wished to stifle, have complained so loudly that in the end it was necessary to let them have their full swing."—*Medical Times & Gazette.*

6. Change of Type.—The change of type theory of disease does not appear to have advanced much in Italy, if we may judge from what we hear concerning the sanguinary therapeutical code still in vigor there. The following, we believe, is a daily example of the practice carried in the way of venesection by our Italian brethren:

"The President opened the sitting by the announcement of the death of General Guaglia, in consequence of the fit of apoplexy with which he was struck in the House the other day, or perhaps of the doctors, who drained the poor aged gentleman to the last drop of his blood—four bleedings in twenty-four hours, three of them after his recovery from the fit."

*A propos* of this incident, we would like to hear how those gentlemen who argue of the truth of this change in type of disease, from the fact that skillful physicians did bleed in other days but don't bleed now, reconcile with their argument this unceasing love of the lancet—to-day just as in other days—by the skillful Italian physician.

"Let me say to those who think that inflammation has changed its type during these last years," says Professor Forgot, "that I believe nothing of the kind. How can this be when nothing around us has changed; when all the local and general phenomena of inflammation remain the same; whilst irritants still preserve the privilege of engendering inflammation, and other remedies of soothing it; when we see at the same time, in the same town, in the same Hospital, at La Charite for example, enlightend practitioners combatting inflammation by opposite means, we are told that inflammation once sthenic has now become asthenic! The fact is that it is now what it always has been, and if anything has changed it is not the Medical constitution, it is the Medical heads that have turned."—*Ib.*

7. GRATUITOUS SERVICES.—The profession has often heard of the abuses of our system of gratuitous hospital advice, and of the manner in which well-to-do persons impose on our public charities. It seems, however, that on the other side of the world impudence is carried even to a greater extent than in this country. A writer on charitable institutions, in the *Australian Medical Journal* for January, 1860, concludes his article with the following paragraph:

"It is no uncommon thing for persons with a rental of two or three hundred a year to send their wives and children to the hospital for relief; that in like manner, store-keepers, who take their £50 or £60 a week, are patients; that we have heard one of the medical officers stating that he has known a man with a rental of £1000 a year attending as an out-patient; that it is a common practice with these very persons to say to their friends, 'why do you pay for medical advice, when you can get it from the hospital for nothing?' There are to be daily seen, in the out-patients' waiting-rooms, persons seeking advice who are better dressed and adorned with more jewelry than the wives and daughters of many of the subscribers, and it has but lately happened that a man who, upon paying a small account as a private patient, had handed a £50 instead of a £5 note, protesting that it was the last money he had in the world, and in a few days after was recognized in one of the wards of the hospital, having obtained admission on the false pretence of poverty."—*British Medical Journal.*

8. Iodine, according to M. Boinet, preserves, cures, strengthens, and modifies the constitution, removes diatheses, and impresses a new energy in the organism. Iodine, according to M. Rilliet, weakens, deteriorates, wastes, destroys, atrophies, and kills?

Last year M. Beau discovered that lead was an excellent remedy for phthisis. M. Broeckx, of Antwerp, has tried the mineral extensively, and has found it worse than useless!

M. Chatin lately informed the French Academy, *a propos* of iodine, that Coindet had by its use reduced so many women to the condition of Amazons and had brought such a number of men into the state described by M. Ricord under the term "haricocle," that he dare not show himself in the streets of Geneva, through dread of suffering the martyrdom of St. Stephen.—*Ib.*

9. Medical men are found in every part of the earth; and they do not always confine themselves to ministering to physical maladies. As an example, we may call attention to the fact that at a recent "Missionary Conference" at Liverpool, Dr. Lockhart, of Liverpool, late a medical missionary in China, in an interesting speech, recounted his experience in China. He spoke highly of the system of employing medical gentlemen as missionaries; its success in China had been marked and satisfactory. He had been in the north of China, where the face of a European was almost an object of fear and surprise, but alone he had succeeded by degrees in winning the confidence and affection of the native. Surgery was in a low state in China, so that a properly-qualified medical man soon obtained great influence. At Shanghae he opened a hospital, which was attended by three hundred or four hundred natives—at one time by the wounded pirates, Imperialist soldiers and citizens together. His introduction of vaccination opened the houses of the influential to him to an unexpected degree.—*Medical Times & Gazette.*

10. SEVERE WOUNDS—SLOW DEATH.—The following facts appear to be authenticated by Mr. Surgeon Hill, as well as by non-medical witnesses in the case of the murder of Jno. Groundwater, by Butterworth and Jenison, in 1794; they struck him with their shovels three blows upon the skull causing a double handfull of his brains to fall out; they then struck with the same upon the neck, intending to severe his head from the body. He lived eighteen hours afterwards; the pulse was strong and the respiration continuous the whole eighteen hours. *Celebrated Trials.* Lond. 1825, vol. v, 282.)

11. THE NIGER EXPEDITION.—Dr. M'Cormac, of Belfast (in *Edin. Medical & Surgical Journal*, October, 1845), says, in an article on the Niger Expedition: "Of 158 negroes employed in the Niger expedition, only eleven were attacked with fever—all recovered. Of 145 whites, 130 contracted the fever, 40 perished, 15 escaped from it.

12. SCROFULA.—Dr. Gregory, of Edinburgh, asserted as his belief that there was not a single family in Great Britain in which scrofula did not exist.

13. CRIMINALS CONDEMNED TO DEATH.—In the *Celebrated Trials* (6 vols. Lond. 1825), a number of cases are recorded of person condemned to death who died on the way to execution from remorse, sorrow and fear; while others hailed death with pleasure. It is a remarkable fact often mentioned in this work, that criminals frequently sleep profoundly the night before their execution.

14. THE TORTURE IN THE CRIMINAL JURISPRUDENCE OF THE 19TH CENTURY.—In the work last mentioned, it is shown that, in 1806, the British Governor of Trinidad, repeatedly inflicted the Torture on Louisa Calderdon, a girl aged 11 years, suspected of robbery. (Vol. vi. p. 1).

15. Consolation for Sciolists.—A physician once said to Barthez, "Your book is much too difficult to be understood." "Patience," replied Barthez, "I am preparing an edition which will be so clear that every ass will be able to drink from it."

16. Systems.—The celebrated Bordeau said: "I was dogmatic at 20, an observer at 30, an empiric at 40, and now at 50, I no longer have any systems."—*N. O. Med. & Surgical Journal.*

